# Screening for Bioactive Metabolites in Plant Extracts Modulating Glucose Uptake and Fat Accumulation

**DOI:** 10.1155/2014/156398

**Published:** 2014-08-28

**Authors:** Rime B. El-Houri, Dorota Kotowska, Louise C. B. Olsen, Sumangala Bhattacharya, Lars P. Christensen, Kai Grevsen, Niels Oksbjerg, Nils Færgeman, Karsten Kristiansen, Kathrine B. Christensen

**Affiliations:** ^1^Department of Chemical Engineering, Biotechnology and Environmental Technology, University of Southern Denmark, Campusvej 55, 5230 Odense M, Denmark; ^2^Department of Biology, University of Copenhagen, Ole Maaløes Vej 5, 2200 Copenhagen N, Denmark; ^3^Department of Biochemistry and Molecular Biology, University of Southern Denmark, Campusvej 55, 5230 Odense M, Denmark; ^4^Department of Food Science, Aarhus University, Blichers Allé, P.O. Box 50, 8830 Tjele, Denmark; ^5^Department of Food Science, Aarhus University, Kirstinebjergvej 10, 5792 Aarslev, Denmark

## Abstract

Dichloromethane and methanol extracts of seven different food and medicinal plants were tested in a screening platform for identification of extracts with potential bioactivity related to insulin-dependent glucose uptake and fat accumulation. The screening platform included a series of* in vitro *bioassays, peroxisome proliferator-activated receptor (PPAR) *γ*-mediated transactivation, adipocyte differentiation of 3T3-L1 cell cultures, and glucose uptake in both 3T3-L1 adipocytes and primary porcine myotubes, as well as one* in vivo* bioassay, fat accumulation in the nematode* Caenorhabditis elegans*. We found that dichloromethane extracts of aerial parts of golden root (*Rhodiola rosea*) and common elder (*Sambucus nigra*) as well as the dichloromethane extracts of thyme (*Thymus vulgaris*) and carrot (*Daucus carota*) were able to stimulate insulin-dependent glucose uptake in both adipocytes and myotubes while weekly activating PPAR*γ* without promoting adipocyte differentiation. In addition, these extracts were able to decrease fat accumulation in* C. elegans*. Methanol extracts of summer savory (*Satureja hortensis*), common elder, and broccoli (*Brassica oleracea*) enhanced glucose uptake in myotubes but were not able to activate PPAR*γ*, indicating a PPAR*γ*-independent effect on glucose uptake.

## 1. Introduction

Type 2 diabetes is an increasing health problem affecting populations worldwide. Insulin resistance is the prediabetic state where insulin sensitive tissues such as muscles, adipocytes, and liver show reduced sensitivity towards insulin and a decreased glucose uptake (GU), which leads to elevated blood-glucose levels [1]. In the late 1990s, thiazolidinediones (TZDs) were introduced as new effective oral insulin sensitizing drugs for management of type 2 diabetes but due to severe side effects such as increased water retention, weight gain, heart enlargement, and hepatotoxicity most TZDs have been withdrawn from the market [2]. Thus there is a need for new insulin sensitizing drugs with fewer side effects.

TZDs such as rosiglitazone (Rosi) act as full agonists of peroxisome proliferator-activated receptor gamma (PPAR*γ*). Activation of PPAR*γ* by agonists leads to a conformational change in the ligand-binding domain (LBD). This process alters the transcription of several target genes involved in carbohydrate and lipid metabolism [3]. Depending on the ligands they can induce different sets of genes as a result of differential recruitment of cofactors [4, 5]. Recruitment of some cofactors leads to increased lipid storage and decreased energy expenditure, whereas recruitment of others increases insulin-stimulated GU, glucose metabolism, and energy expenditure [6, 7]. The activation of PPAR*γ* by TZDs leads to the elimination of free fatty acids from circulation by promoting their cellular uptake, their storage within adipocytes, and increase of the plasma concentration of certain hormones such as adiponectin, all factors that are known to improve insulin sensitivity [5, 8, 9]. The unwanted side effects of TZDs have been linked to the behavior of TZDs as full agonists of PPAR*γ* [10]. Partial PPAR*γ* agonists are compounds with diminished agonist efficacy that maintain the antidiabetic effect of full agonists but do not induce the same magnitude of side effects as these because they recruit a different set of cofactors compared to full agonists [3, 4, 11]. Consequently, the search for promising PPAR*γ* agonist with an improved mechanism of action is an important objective in the search for new insulin sensitizing drugs.

Over 1000 plant species have been estimated to exhibit antidiabetic effects, and therefore plants are considered a promising source of natural products for novel potentially antidiabetic compounds [12]. Different groups of secondary metabolites have been identified as hypoglycemic agents, for example, terpenoids, alkaloids, and flavonoids [13]. In this study, plants were selected either for their known antidiabetic effects or for their content of metabolites with hypoglycemic activity [14–20]. The objectives of the present study were to examine the effects of plant extracts on PPAR*γ* transactivation, GU in adipocytes and primary myotube cultures, and fat accumulation in* Caenorhabditis elegans* (*C. elegans*) in order to identify promising sources of natural products with an effect on type 2 diabetes.

Christensen et al. [18] previously published results of a screening of 24 plant species using a platform where extracts were screened for their ability to activate PPARs, including PPAR*γ*. Based on the obtained results, the extracts were then tested for stimulation of adipocyte differentiation and effect on GU in adipocytes. The present study used a similar platform but was extended by including assay for GU in muscle stem cell derived myotube cultures, as muscles are one of the most important tissues for GU. The nematode* C. elegans* was used afterwards as a model organism to evaluate the effects of the extracts on fat storage* in vivo*. Thus the applied bioassays in the present study represent an improved platform to evaluate the potential antidiabetic effect of plant extracts.

## 2. Materials and Methods

### 2.1. Plant Material and Extracts

All plant species used in this study (Table 1), except common elder, were cultivated at Department of Food Science, Aarhus University, Aarslev, Denmark. Elder was cultivated at Holunderhof Helle, Thumby, Germany. Carrots and golden root were used fresh; all other plant materials were frozen (−22°C) directly after harvest. All plant materials were blended before extraction and subjected to a two-step sequential extraction procedure with first dichloromethane (DCM) and then methanol (MeOH) (HPLC-grade, Sigma-Aldrich, Germany). 5 mL solvent/g of plant material was used for extractions. The plant material was soaked in each solvent for 24 h at 5°C in the dark with occasional shaking. Extracts were filtered using filter paper with a pore size of 20–25 *μ*m and dried under vacuum (40°C). The described extraction procedure has previously been shown to produce plant extracts with potential antidiabetic effect containing both lipophilic and hydrophilic bioactive compounds [18–20]. Water was not used in the sequential extraction step because we have not previously been able to detect significant bioactivity in water extracts in a sequential extraction procedure and secondly because potential bioactive compounds present in water extracts are too hydrophilic to have any significant bioavailability. A 100 mg/mL sample in dimethylsulfoxide (DMSO) was prepared for each extract and was used as stock solution for the preparation of individual test solutions.

### 2.2. PPAR*γ* Transactivation Assay

Mouse embryonic fibroblasts [21] were trypsinized after reaching 70% confluence and transfected using Metafectene Easy (Biontex Laboratories, Germany) according to the manufacturer's recommendations. For each well (in a 96-well plate) a total of 0.05 *µ*g of DNA were used (0.015 *µ*g pM-hPPAR*γ*-LBD + 0.03 *µ*g Gal4-responsive luciferase reporter + 0.0025 *µ*g pRL-CMV normalization vector). Dulbecco's modified Eagle's medium (DMEM), media containing vehicle (0.1% DMSO), positive control (1 *μ*M Rosi), or extract dissolved in DMSO was added to the cells after 6 h of transfection. Cells were washed with phosphate buffered saline (PBS) after 18 h of transfection and lysed with lysis buffer (0.2 M KH_2_PO_4_, 0.2 M K_2_HPO_4_, 0.4% Triton N-101, 100 *µ*M phenylacetic acid, and 100 *µ*M oxalic acid). Photinus and Renilla luciferase activities were measured directly in the plate as previously described [20]. Photinus activities were normalized to corresponding Renilla values to compensate for differences in transfection efficiency. Results are presented as the mean ± SD.

### 2.3. Cell Culture Study

3T3-L1 preadipocytes were cultured in DMEM with 10% foetal calf serum (FCS) supplemented with 1% penicillin/streptomycin at 37°C in humidified 95% air and 5% CO_2_. At day two postconfluence (designated day 0) cells were induced to differentiate with 500 *μ*M isobutyl-methylxanthine, 1 *μ*M dexamethasone, 167 nM insulin (MDI protocol), or with 1 *μ*M dexamethasone and 167 nM insulin (DI protocol). For both protocols, the medium was replaced on day 2 with DMEM supplemented with 10% FCS, 1% penicillin/streptomycin, and 167 nM insulin, and thereafter every second day with DMEM supplemented with 10% FCS and 1% penicillin/streptomycin. Vehicle cells were treated with 0.1% DMSO (v/v) equal to the DMSO concentration in the medium supplemented with extracts.

### 2.4. Glucose Uptake in 3T3-L1 Cell Cultures

Cells were seeded on 96-well plates and differentiated according to the MDI protocol till day +8. At day +8 cells were fed with designated medium supplemented with DMSO, Rosi, or extract, and a GU assay was performed 48 h later. Cells were washed with 200 *µ*L/well PBS (pH 7.2, 1 mM CaCl_2_, and 1 mM MgSO_4_), subsequently with 200 *µ*L/well DMEM (1 g/L glucose), and finally incubated in 200 *µ*L/well of the same medium for 2 h in the incubator at 37°C and 10% CO_2_. Cells were then washed with 200 *µ*L/well Krebs-Ringer-Hepes buffer (KRHB, pH 7.4) and incubated with 50 *µ*L/well KRHB for 30 min using the same conditions as described above. 50 *µ*L/well KRHB containing double amount of the designated concentrations of insulin was added, and incubation was continued for exactly 15 min. GU was initiated by addition of 50 *µ*L/well KRHB (3.0 mM glucose, 0.15 *µ*L [14C] 2-deoxy-D-glucose (5 mCi/L) yielding a final concentration of 1.0 mM glucose). 2-Deoxy-D-glucose was used because this compound is not metabolized by cells. The cells were incubated for exactly 15 min in the incubator at 37°C and 10% CO_2_. Then 50 *µ*L/well Quench buffer (800 mM D-glucose, 50 mM 4-(2-hydroxyethyl)-1-piperazineethanesulfonic acid (HEPES), pH 7.5, and 262 mM NaCl) was added. Cells were washed three times in 200 *µ*L/well ice-cold PBS and lysed in 200 *µ*L/well 1% sodium dodecyl sulphate by shaking for 2 h. Radioactivity in the lysates was determined by scintillation counting. GU was determined in eight parallel wells for each extract and for each insulin concentration. The results are presented as the mean ± SD.

### 2.5. Preparation of Myotube Cultures

Satellite cells were isolated from semimembranous muscles of female pigs weighing approximately 12 Kg, as reported by Theil et al. [22], and stored in liquid nitrogen until used. To prepare myotube cultures, the cells were seeded on Matrigel matrix (BD Biosciences) coated (1 : 50 v/v) 48-well plates for GU assay. Cells were proliferated in porcine growth medium (PGM) consisting of 10% FCS, 10% horse serum, and 80% DMEM with 25 mM glucose and antibiotics (100 IU/mL penicillin, 100 IU/mL streptomycin sulfate, 3 *µ*g/mL amphotericin B, and 20 *µ*g/mL gentamycin). The cells were grown in PGM until they reached approximately 80% confluence in a CO_2_-related humidified incubator (95% air, 5% CO_2_ at 37°C). The cells were allowed to proliferate to 100% confluence in media containing DMEM (7 mM glucose), 10% FCS, and antibiotics for 24 h and then differentiated into myotubes by incubating with differentiation media (DMEM with 7 mM glucose, 5% FCS, antibiotics, and 1 *µ*M cytosine arabinoside) for at least 48 h.

### 2.6. Glucose Uptake in Porcine Myotube Cell Cultures

Differentiated myotubes were treated with serum free media (DMEM with 7 mM glucose, antibiotics, and 1 *µ*M cytosine arabinoside) overnight, followed by incubation with treatments for 1 h. The myotubes were then washed thrice with HEPES buffered saline (20 mM HEPES, 140 mM NaCl, 5 mM KCl, 2.5 mM MgSO_4_, and 1 mM CaCl_2_, adjusted to pH 7.4 with 2 M NaOH) and incubated with 250 *µ*L of 0.1 mM 2-deoxy-[^3^H]-D-glucose per well for 30 min. Again 2-deoxy-D-glucose was used as it is not metabolized by the cells. Myotubes were then quickly washed thrice with 500 *µ*L ice-cold PBS per well and the cells lysed by adding 250 *µ*L of 0.05 M NaOH (37°C) per well and were placed on a shaking board for 30 min. The cell lysate was transferred to a scintillation tube, mixed with scintillation liquid (Ultima Gold, PerkinElmer Inc.) in 1 : 10 ratio, and counted in a Win spectral, 1414 liquid scintillation counter (PerkinElmer). For each experiment, satellite cells from 3 pigs were used, with 6 replicates per pig.

### 2.7. Fat Accumulation in* C. elegans*

Worms were maintained as described by Sulston and Hodgkin [23]. The wild-type reference strain was the* C. elegans* Bristol variety strain, N2. Nile red was dissolved in acetone (500 mg/mL) and added to molten nematode growth medium (NGM) (~55°C) to a final concentration of 0.05 mg/mL. NGM was poured into 24-well plates (1 mL/well). Wells were seeded with 40 *µ*L uracil auxotroph* E. coli* strain OP50 in 2 × yeast (yeast tryptone, YT) medium per well and, when dried, 25 *µ*L M9 salt solution (3 g KH_2_PO_4_, 6 g Na_2_HPO_4_, 5 g NaCl, 1 mL 1 M MgSO_4_, and H_2_O to 1 L; sterilized by autoclaving) and 10 *µ*L DMSO or extract were added. Crude extracts were diluted to stocks of 20 mg/mL in DMSO; thus they were tested at a final concentration of 200 *µ*g/mL. Worms were synchronized by standard bleaching protocol [24] followed by hatching to L1 in M9 supplemented with cholesterol overnight. L1 larvae were put on the plates and grown for 46 h at 20°C until mid-L4 stage. Worms were then mounted in a drop of 10 mM tetramisole (T1512, Sigma-Aldrich) atop 2% agarose pads laid on a microscopy glass slide and then overlaid with a cover slip. Fluorescence microscopy (rhodamine channel) was done using a Leica DMI6000 B microscope equipped with an Olympus DP71 camera. Images were captured using Visiopharm Integrator System software (Visiopharm, Denmark). For nile red staining, all worms were photographed using 200x magnification, maximum fluorescence intensity, and a fixed exposure time of 30 ms. Images were quantified using ImageJ (image processing and analysis in Java; http://rsbweb.nih.gov/ij/).All images in each experiment were background corrected by subtracting the same value from each pixel. Staining levels were quantified as the integrated fluorescence density. The results are presented as means ± SD and normalized to DMSO treated worms.

### 2.8. Statistical Analysis

For studies on adipocytes *t*-tests were done for assessment of significance. For experiments on porcine myotubes, statistical analysis of data was conducted using the mixed model procedure of SAS statistical programming software (ver. 9.2, SAS Institute Inc., Cary, NC). The model consisted of the fixed effects of treatments (concentration of extracts and extract type) and their interactions. Experiments, replicates, and pigs within treatments were included as well as their appropriate interactions as random effects. Least square means (LSMeans) ± standard errors of LSMeans (SEM) were calculated and effects of extract concentrations and extract types are presented relatively to the control (DMSO). For studies on* C. elegans*, statistical analyses were performed in GraphPad Prism by subjecting the data to a one-way ANOVA followed by Dunnett's multiple comparison test.

## 3. Results and Discussion

In this study several bioassays were used to test the following plant species:* Brassica oleracea* var.* italica* (broccoli) and var.* sabellica* (kale),* Daucus carota* (carrot),* Rhodiola rosea* (golden root),* Satureja hortensis* (savory),* Sambucus nigra* (elder), and* Thymus vulgaris* (thyme) (Table 1) for their potential bioactivities related to glucose homeostasis. The plants were exposed to a two-step sequential extraction procedure using DCM and MeOH to achieve an initial crude separation of the plant metabolites based on their polarity.

The screening platform previously described by Christensen et al. [18] that enables the identification of potential partial PPAR*γ* agonists with little or no promotion of adipogenesis was applied in this study as well. The platform consists of different bioassays and firstly tests the ability of the plant extracts to activate PPAR*γ* in transfected cells. If activation of PPAR*γ* is present, the adipogenic potential of the extract is determined* in vitro* by an adipocyte differentiation assay. If no significant stimulation of adipocyte differentiation is observed, the ability of the extract to enhance insulin-dependent GU in adipocytes is tested. Muscles are one of the essential organs for glucose metabolism and furthermore are strongly affected by insulin resistance and, hence, the present screening study also included a bioassay, testing the effect of the plant extracts on insulin-stimulated GU in porcine myotubes. Plant extracts exhibiting the modest activation of PPAR*γ*, no (or minimal) stimulation of adipocyte differentiation, and ability to enhance insulin-dependent GU in adipocytes and myotubes are therefore considered as a good source for the isolation and identification of potential bioactive constituents with antidiabetic effect.

To supplement the studies on glucose homeostasis* in vitro*, the extracts were also tested for their effect on fat accumulation in the worm* C*.* elegans*, which has been shown to serve as a useful model for the genetic analyses of energy homeostasis and fat storage pathways in a whole organism [25]. The short life cycle of* C. elegans* reduces the time needed for an experimental cycle and therefore it is advantageous as an* in vivo* model for fast screening.

Sixteen extracts from seven plant species were tested in the bioassays (Table 2). Seven extracts were able to activate PPAR*γ* and were then tested for their adipogenic potential in the* in vitro* adipocyte differentiation assay. No stimulation of adipocyte differentiation was observed for any of these extracts except the DCM extract of broccoli, which caused cell death (Table 2). Broccoli is known for its high content of glucosinolates that can be hydrolyzed to the lipophilic isothiocyanates by the enzyme myrosinase. Isothiocyanates have previously been reported to cause antiproliferation and apoptosis [26] and thus the cytotoxic effect of this extract may have been caused by high levels of isothiocyanates. Five of the extracts able to activate PPAR*γ* without stimulation of adipocyte differentiation were also able to increase insulin-stimulated GU in adipocytes (Table 2). Four of the five extracts were able to stimulate GU in adipocytes and to enhance insulin-stimulated GU in primary porcine myotubes. The nine extracts not able to activate PPAR*γ* were included in the test for effect on GU in myotubes and three of these (MeOH extracts of broccoli, elder, and savory) were able to enhance GU significantly. All sixteen plant extracts were also tested in the* in vivo* assay for fat accumulation in* C. elegans* and of these thirteen were able to decrease fat accumulation including the four extracts that were able to activate PPAR*γ* as well as positively affecting GU in both adipocytes and myotubes.

**Figure 1:**
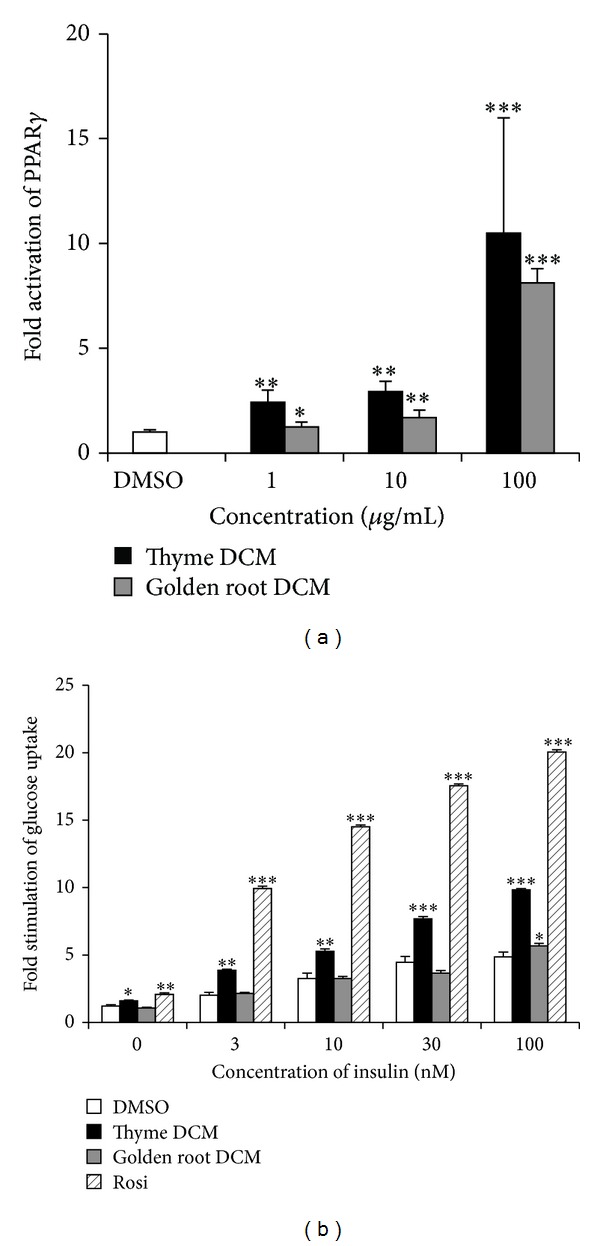
Effect of DCM extracts of golden root (*Rhodiola rosea*) and thyme (*Thymus vulgaris*) on PPAR*γ* transactivation (a) and glucose uptake (GU) in adipocytes (b). DMSO (vehicle, set to 1.00) and results were normalized to this. For PPAR*γ*, positive control was rosiglitazone (Rosi, fold activation 79.7 relative to DMSO) and test concentrations of extracts were 1, 10, and 100 *μ*g/mL (a). The GU test was performed at different concentrations of insulin (0, 3, 10, 30, and 100 nM), while the extract concentration was kept at 100 *μ*g/mL (b). Plotted values are least square means ± SD of mean of 3 replicates. *P < 0.05, **P < 0.01, and ***P < 0.001 indicate significance relative to DMSO.

**Figure 2:**
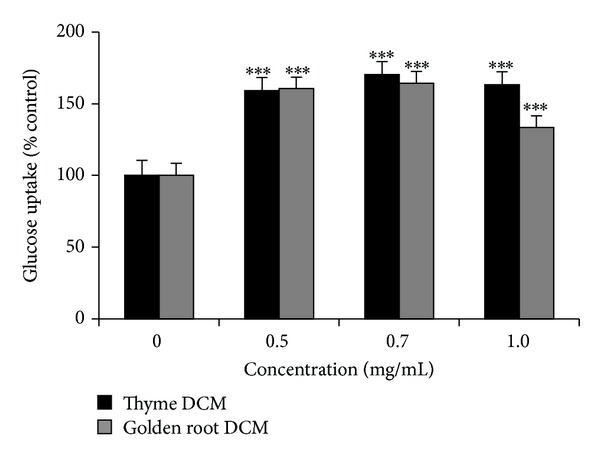
Effect of thyme (*Thymus vulgaris*) and golden root (*Rhodiola rosea*) DCM extracts on insulin-stimulated glucose uptake (GU) in primary porcine myotube cultures. The concentration of insulin was kept at 750 pM, while the concentration of the extract was increasing from 0 till 1 mg/mL. GU is given as percent of the control (set to 100). Number of pigs used was 3 and number of replicates per pig was 6. The plotted values are least square means ± SD of mean. *P < 0.05, **P < 0.01, and ***P < 0.001 indicate significance relative to DMSO.

Two species of the Lamiaceae family (thyme and savory) were tested and the DCM extracts of both were able to activate PPAR*γ* more than 10-fold relative to the vehicle (DMSO) without stimulation of adipocyte differentiation. Carvacrol, a major component in thyme, has previously been reported to activate PPAR*γ* [17] and this might explain some of the observed bioactivity. Results for thyme are illustrated in Figure 1 and correspond to those obtained by Christensen et al. for both the DCM and the MeOH extract [18]. Stimulation of GU in adipocytes (Figure 1) and myotubes (Figure 2) by the DCM extract of thyme was significant compared to the control. GU-related activity in adipocytes was reported previously [18] for both thyme and savory extracts, while no records are available on their activity in myotubes. The stimulation of GU in myotubes by the DCM extract of thyme (Figure 2) was dose-dependent until 0.7 mg/mL and hereafter activity slightly decreased at the maximum concentration of 1 mg/mL. This decrease in activity might be explained as a cytotoxic effect of the extract at high concentration, which was also observed by Rozman et al. [27], who tested several compounds from thyme including carvacrol for their toxicity against insects.

**Figure 3:**
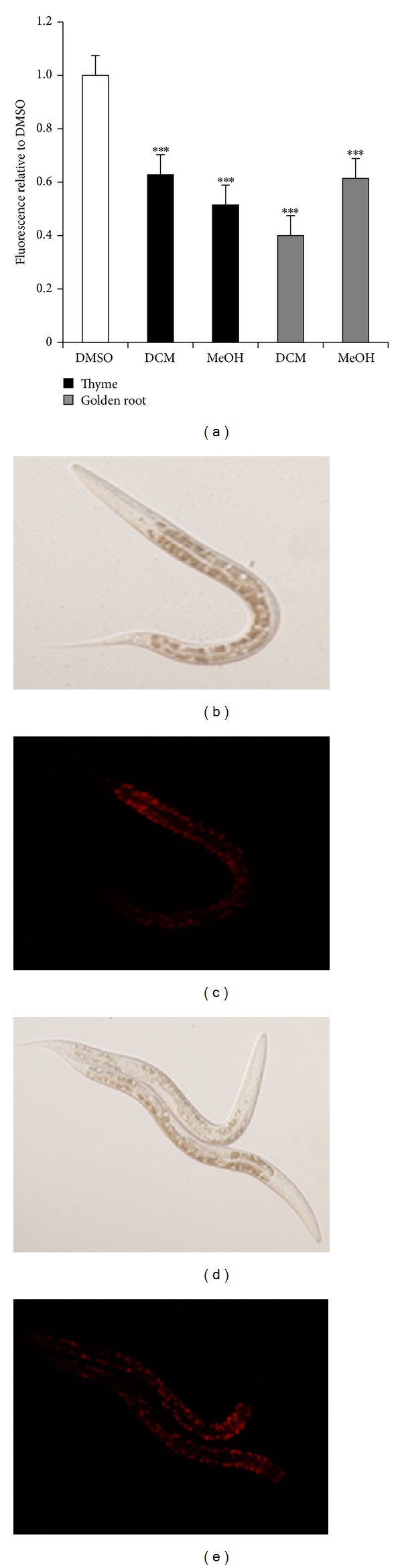
Effect of DCM extracts of golden root (*Rhodiola rosea*) and thyme (*Thymus vulgaris*) on fat accumulation in* C. elegans.* Activation by DMSO (vehicle set to 1.00) and results are given as fluorescence levels relative to this (a). All extracts were tested at 200 *μ*g/mL. Lipid stores in* C. elegans* were stained with nile red. Differential interference microscopy and nile red images (rhodamine channel) of L4 staged wild-type worms treated with either vehicle (b and c) or 200 *µ*g/mL DCM extract of thyme (d and e) are shown. Fluorescence levels are shown as normalized means ± normalized SEM. *P < 0.05, **P < 0.01, and ***P < 0.001 indicate significance relative to DMSO.

DCM extracts of both aerial parts and roots of golden root were tested in this study. Only the DCM extract of aerial parts activated PPAR*γ* without stimulating adipocyte differentiation (Figure 1) and stimulated GU in adipocytes (Figure 1) and myotube cell cultures (Figure 2) as well. Golden root is known for a high content of phenolic glycosides, for example, salidroside that has previously been shown to have an effect on carbohydrate metabolism and adipocyte differentiation [16]. However, these phenolic glycosides are much more soluble in MeOH than in DCM, and as the MeOH extracts of golden root showed no activity in the bioassays this indicates that the active compounds are not phenolic glycosides but other less polar compounds. In* C. elegans*, both DCM and MeOH extracts of golden root were able to suppress fat accumulation significantly compared to the vehicle (Figure 3).

Christensen et al. [18] tested four different extracts of elderflowers (hexane, DCM, MeOH, and ethyl acetate) to determine their PPAR-related activities and all activated PPAR*γ* along with PPAR*α* and *δ*. In the present study, we were able to confirm that the DCM extract activates PPAR*γ* but not the MeOH. This could be due to the different methods of extraction applied as Christensen et al. did a four-step sequential extraction, while only a two-step procedure was applied here. The DCM extract of elderflowers stimulated GU in both adipocytes and myotubes in a dose-dependent manner corresponding with Christensen et al. [18]. The MeOH extract of elderflowers was not tested for effect on GU in adipocytes as it was unable to activate PPAR*γ* and previous results showed it to be inactive [18]; however it was found to be able to increase GU in myotube cell cultures (Table 2).

## 4. Conclusions

The present screening provides valuable information on the bioactivities of different plant species in relation to aspects of glucose homeostasis and shows that plant foods in human nutrition contain compounds that may help to improve glucose homeostasis. Some extracts were able to activate PPAR*γ* and also enhance GU in both adipocytes and myotubes. Other extracts were unable to activate PPAR*γ* but were still able to stimulate GU in myotubes. Future investigations using bioassay-guided chromatographic fractionations may lead to identification of specific compounds from these plant extracts responsible for the observed bioactivities and may explain their mechanism of action in relation to GU both* in vitro* and* in vivo*.

## Figures and Tables

**Table 1:** The seven different plant species tested in the bioassays of this screening study.

Botanical name	Common name	Family	Part of plant
*Brassica oleracea *var.* italica *	Broccoli	Brassicaceae	Aerial parts
*B. oleracea *var.* sabellica *	Kale	Brassicaceae	Aerial parts
*Daucus carota *	Carrot (cv purple haze)	Apiaceae	Roots
*Rhodiola rosea *	Golden root	Crassulaceae	Flowers/roots
*Sambucus nigra *	Common elder	Caprifoliaceae	Flowers
*Satureja hortensis *	Savory, summer	Lamiaceae	Aerial parts
*Thymus vulgaris *	Thyme	Lamiaceae	Aerial parts

**Table 2:** Results obtained for the plant extracts in the bioassays (test concentration 100 *μ*g/mL) of the screening platform. Results are given as either (+) for activation/stimulation or (−) for no activation/stimulation. For *C. elegans* (+) means reduction of fat accumulation (acc.). Extracts not tested are indicated by (nt), cytotoxic effects are indicated by (†), and glucose uptake is indicated as (GU).

Plant species	Extract	PPAR*γ* transactivation	Adipocyte differentiation	GU in adipocytes	GU in myotubes	Fat acc. in *C. elegans *
Broccoli	DCM	+	†	nt	−	+
	MeOH	−	nt	nt	+	−

Kale	DCM	−	nt	nt	−	+
	MeOH	−	nt	nt	−	+

Carrot	DCM	+	−	+	+	+
	MeOH	−	nt	nt	−	−

Golden root	^ a^DCM	+	−	+	+	+
	^ a^MeOH	−	nt	nt	−	+
	^ b^DCM	−	−	+	+	+
	^ b^MeOH	−	nt	nt	−	+

Elder	DCM	+	−	+	+	+
	MeOH	−	nt	nt	+	+

Savory	DCM	+	−	+	−	+
	MeOH	−	nt	nt	+	−

Thyme	DCM	+	−	+	+	+
	MeOH	+	−	nt	+	+

^a^Aerial parts; ^b^roots.

## Abbreviations

DCM: Dichloromethane
DMEM: Dulbecco's modified Eagle's medium
DMSO: Dimethylsulfoxide
FCS: Foetal calf serum
GU: Glucose uptake
HEPES: 4-(2-Hydroxyethyl)-1-piperazineethanesulfonic acid
KRHB: Krebs-Ringer-Hepes buffer
LBD: Ligand-binding domain
MeOH: Methanol
NGM: Nematode growth medium
PBS: Phosphate buffered saline
PGM: Porcine growth medium
PPAR: Peroxisome proliferator-activated receptor
Rosi: Rosiglitazone
TZD: Thiazolidinedione.
